# microRNAs in the Regulation of Melanogenesis

**DOI:** 10.3390/ijms22116104

**Published:** 2021-06-05

**Authors:** Yekatsiaryna Hushcha, Irene Blo, Lucia Oton-Gonzalez, Giulia Di Mauro, Fernanda Martini, Mauro Tognon, Monica De Mattei

**Affiliations:** 1 Morgan S.r.l, R&D, 36050 Monteviale, VI, Italy; yekatsiaryna.hushcha@gmail.com or; 2 Department of Medical Sciences, Section of Experimental Medicine, School of Medicine, University of Ferrara, 64b, Fossato di Mortara Street, 44121 Ferrara, Italy; irene01.blo@edu.unife.it (I.B.); lucia.otongonzalez@unife.it (L.O.-G.); dmrgli@unife.it (G.D.M.); fernanda.martini@unife.it (F.M.); mauro.tognon@unife.it (M.T.); 3 Laboratory for Technologies of Advanced Therapies (LTTA), University of Ferrara, 44121 Ferrara, Italy

**Keywords:** melanocyte, melanogenesis, microRNA, skin pigmentation

## Abstract

Melanogenesis is the process leading to the synthesis of melanin, the main substance that influences skin color and plays a pivotal role against UV damage. Altered melanogenesis is observed in several pigmentation disorders. Melanogenesis occurs in specialized cells called melanocytes, physically and functionally related by means of autocrine and paracrine interplay to other skin cell types. Several external and internal factors control melanin biosynthesis and operate through different intracellular signaling pathways, which finally leads to the regulation of microphthalmia-associated transcription factor (*MITF*), the key transcription factor involved in melanogenesis and the expression of the main melanogenic enzymes, including TYR, TYRP-1, and TYRP-2. Epigenetic factors, including microRNAs (miRNAs), are involved in melanogenesis regulation. miRNAs are small, single-stranded, non-coding RNAs, of approximately 22 nucleotides in length, which control cell behavior by regulating gene expression, mainly by binding the 3′ untranslated region (3′-UTR) of target mRNAs. This review collects data on the miRNAs involved in melanogenesis and how these miRNAs can modulate target gene expression. Bringing to light the biological function of miRNAs could lead to a wider understanding of epigenetic melanogenesis regulation and its dysregulation. This knowledge may constitute the basis for developing innovative treatment approaches for pigmentation dysregulation.

## 1. Introduction

Skin represents the primary line of defense against environmental stressors, including chemical stimuli, microbial insults, allergens, and ultraviolet (UV) radiation. Protection from UV rays is essentially based on melanogenesis, the process leading to the synthesis of pigments called melanin, the main substance that influences skin color. Melanin protects the skin from harmful UV rays, as it can absorb UV and visible light and shows antioxidative and radical scavenging abilities, limiting UV-induced effects on cellular macromolecules, mainly DNA, thus protecting cells from genotoxic damage [1]. Therefore, reduced melanogenesis is also a major risk factor for melanoma and other skin cancers [2,3]. Nevertheless, increased melanogenesis and melanin accumulation is associated with hyperpigmentation disorders [4,5]. Skin hyperpigmentation, often associated with aging, hormonal changes, and UVB, is very common in clinical dermatology and includes dermal conditions such as melasma, chloasma, freckles, age spots, and sunspots [5]. In addition, both hyperpigmentation and hypopigmentation, as observed in vitiligo lesions, are frequently the consequence of inflammation, induced by skin stressors [6]. Indeed, the modulation of skin pigmentation still represents a challenge in treating dermatological disorders, despite several studies having investigated potential cures [7,8]. Melanin is produced by highly specialized cells called melanocytes that are in strict contact with other skin cells, especially keratinocytes. The process of skin coloration consists of melanin biosynthesis and the translocation of melanosomes, small organelles containing melanin, from melanocytes into epidermal keratinocytes [6,7,9].

Melanogenesis is a very complex process which includes the development, survival, and differentiation of melanocytes. It involves more than 150 genes and several signaling pathways that operate both at transcriptional and post-transcriptional levels in regulating the main melanogenic molecular players. These include transcription factors, enzymes, and regulatory molecules produced by melanocytes, as well as other skin cells including keratinocytes, dermal fibroblasts, and inflammatory and endocrine cells [6,7,10,11].

Altered gene expression and melanogenic regulatory factor activities are involved in melanogenesis dysfunction [4,11]. Increasingly, studies indicate that gene expression is also influenced by several epigenetic events, including chromatin modification, DNA methylation, and non-coding RNA classes such as long non-coding RNAs (lncRNAs) and microRNAs (miRNAs) [12,13,14,15]. Indeed, the role of miRNAs in melanogenesis has been widely investigated. The aim of this review is to collect recent information concerning the role of miRNAs in melanogenesis and its dysregulation. Studies investigating the role of miRNA in modulating tumor development and growth or metastasis in skin cancers were not included and reviewed by others [16]. Analysis of the biological function of miRNAs will allow for a better understanding of the molecular events involved in regulating skin pigmentation. We suggest that this knowledge may lead to the development of innovative diagnostic tools as well as treatment approaches for pigmentation-related disorders.

## 2. Melanin Biosynthesis

Melanin synthesis occurs in highly specialized cells called melanocytes, localized in the basal layer of the epidermis and hair follicles [17,18]. Melanocytes consist of several ramifications called dendrites that end in keratinocytes, and each melanocyte is strictly connected to more than 30 keratinocytes, constituting the melanogenic unit [19]. During melanogenesis, a series of sequential reactions synthesize melanin, which is translocated into neighboring keratinocytes by means of melanosomes [6,7,9].

**Figure 1:**
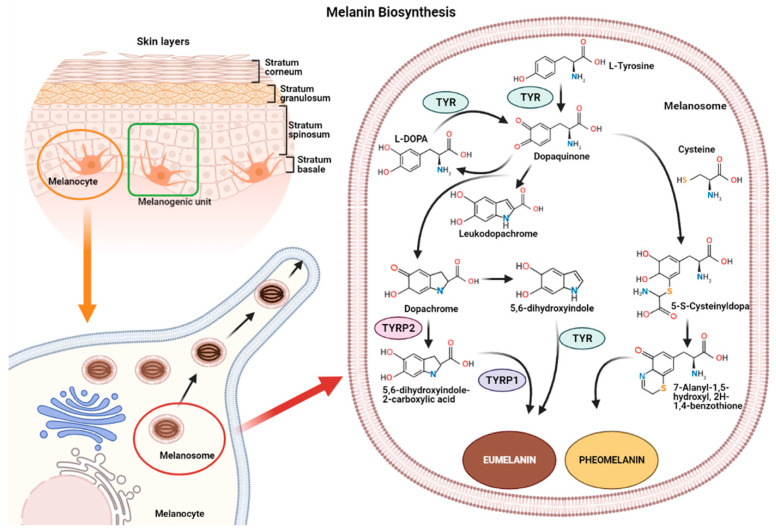
Representation of the melanogenic unit and melanin synthesis in melanosomes (**left**). Schematic representation of eumelanin and pheomelanin biosynthetic pathways (**right**).

Melanin production is controlled by several enzymes including tyrosinase (TYR), tyrosine hydroxylase I (THI), and phenylalanine hydroxylase (PAH) in the initiation phase of melanin synthesis. Tyrosinase-associated protein 1 (TYRP-1) and tyrosinase-associated protein 2 (TYRP-2), also called dopachrome tautomerase (DCT), operate in the later phase [4,20]. TYR is a membrane-bound glycoprotein which plays a key role in the process, as it is considered the rate-limiting enzyme for melanin biosynthesis (Figure 1).

The reaction catalyzed by TYR leads to L-tyrosine being transformed into dopaquinone by oxidation [21]. Dopaquinone is highly reactive and can follow two reaction chains from which eumelanin and pheomelanin originate:

(1) In reactions leading to eumelanin production, dopaquinone undergoes intramolecular cyclization to produce leukodopachrome (cyclodopa). Cyclodopa undergoes redox exchange with another molecule of dopaquinone to form dopachrome and DOPA [21]. The dopachrome downstream process is branched in two ways. The first leads to the formation of 5,6-dihydroxyindole-2-carboxylic acid (DHICA) through TYRP-2 intervention and then into eumelanin by TYRP-1 conversion. The second leads to the conversion of dopachrome into 5,6-dihydroxyindole (DHI) and then into eumelanin involving TYR. At the end of this reaction, black-brownish eumelanin is formed.
(2) In reactions leading to pheomelanin production, in the presence of cystein or glutathione, dopaquinone can be converted into 5-S-cysteinyldopa, or glutathionyldopa, which is then converted into quinoline and finally polymerized into red-yellow pheomelanin.

In the skin, both eumelanin and pheomelanin form complex heteropolymers. The total amount of melanin, as well as the ratio between eumelanin to pheomelanin, are considered to determine skin color. Indeed, the role of the ratio between eumelanin and pheomelanin is still under debate, as many authors indicate that it remains unchanged in various phototypes of *human* skin and, thus, skin color is dependent on total melanin amount [4,22]. Synthesized melanin is collected into melanosomes, which follow a complex maturation process and are transported to keratinocytes along actin filaments in association with motor proteins [4,23].

## 3. Melanogenesis Regulation

**Figure 2:**
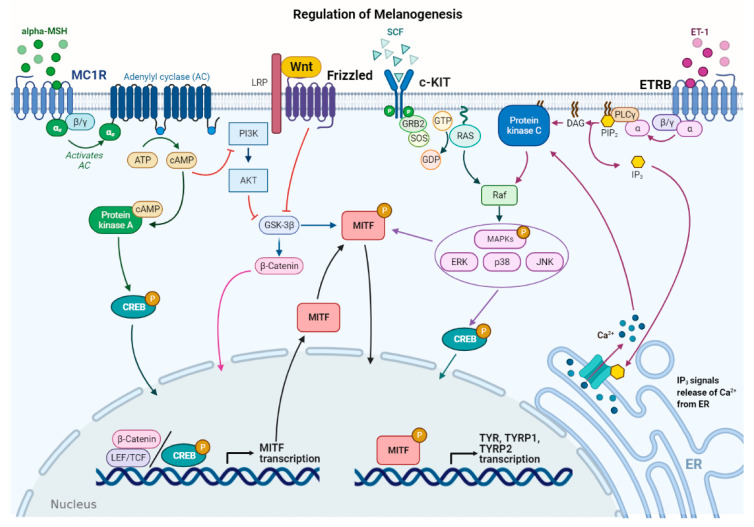
Main signaling pathways involved in melanogenesis regulation. α-melanocyte stimulating hormone (α-MSH) binds to melanocortin 1 receptor (MC1R), which increases cAMP levels, activating PKA and PI3K/AKT pathway. The former phosphorylates CREB protein, promoting *MITF* transcription; the latter interplays with Wnt/β-catenin pathway by phosphorylating GSK-3β, which, in turn, releases β-catenin to promote *MITF* transcription. Stem cell factor (SCF) binds to c-KIT receptor, activating MAPK pathway and phosphorylating CREB. Peptide endothelin 1 (ET-1) binds to its receptor, ETRB, activating PKC and stimulating *MITF* transcription. *MITF* is phosphorylated at the post-transcriptional level to promote transcription of the melanogenic enzymes.

Several stimuli control melanogenesis, including external factors such as UV rays or environmental pollution [11]. Furthermore, a series of endogenous molecules released by melanocytes, keratinocytes, dermal fibroblasts, and immune cells modulate the process paracrinally and autocrinally. These are mainly the α-melanocyte stimulating hormone (α-MSH) and the adrenocorticotropic hormone (ACTH), derived from the cleavage of pro-opiomelanocortin (POMC), from both melanocytes and keratinocytes [11,24]. In addition, stem cell factor (SCF), peptide endothelin 1 (ET-1), hepatocyte growth factor (HGF), keratinocyte growth factor (KGF), basic fibroblast growth factor (bFGF), and inflammatory mediators such as cytokines, prostaglandin E2 (PGE2), and nitric oxide (NO) significantly regulate melanogenesis [25,26]. All these stimuli operate with the activation of several signaling pathways [9,27]. Detailed descriptions of the multiple pathways in melanogenesis have been recently reported by others [6,7,9,11]. Here, the main signaling pathways are briefly summarized (Figure 2).

In melanocytes, signaling pathways regulating melanogenesis operate through membrane receptors with different molecular activities. These receptors include G protein-coupled receptors (GPCRs) such as the melanocortin-1 receptor (MC1R), which is mainly expressed in melanocytes [28], adrenergic receptors, endothelin type B receptor (ETRB), frizzled receptor (FZD), and tyrosine kinase receptors such as tyrosine kinase receptor KIT, bFGF, and HGF receptors [7,29]. Most signaling pathways lead to the regulation of microphthalmia-associated transcription factor (*MITF*), a basic helix–loop–helix leucine zipper (bHLH-ZIP) transcription factor. *MITF* is the dominant transcription factor in melanogenesis as it controls melanocyte development, survival, and proliferation, as well as the steps involved in melanin synthesis [30,31]. *MITF* induces the transcription of the melanogenic genes, including *TYR*, *TYRP-1*, and *TYRP-2*, by binding to the conserved consensus elements of the promoter regions [31]. Moreover, *MITF* regulates several other genes involved in melanogenesis, including those required to control melanosome maturation, traffic, and distribution to keratinocytes [32]. Due to the prominent role of *MITF*, regulation of its expression and activity represents a key event in melanogenesis. At the transcriptional level, *MITF* expression is regulated by several transcriptional factors that bind the *MITF* promoter, including cyclic adenosine monophosphate (cAMP)-response element binding protein (CREB), paired box family of transcription factor 3 (PAX3), sex determining region Y-box 9 and 10 (Sox9, Sox10), and Wnt/β-catenin pathway effector lymphoid enhancer-binding factor 1 (LEF-1) [7,31].

Furthermore, *MITF* activity is regulated at the post-transcriptional level mainly by phosphorylation [33]. *MITF* may be phosphorylated by several kinases such as MAPK, p38, ribosomal S6 kinase (RSK), and GSK3β [7,11,34,35]. *MITF* phosphorylation can favor the recruitment of transcriptional coactivator CBP/P300 of CREB, thus increasing the transcription of TYR, TYRP-1, and TYRP-2 melanogenic enzymes [31]. The MAPK signaling pathway can be activated by several extracellular factors, which operate through tyrosine kinase receptors including SCF, a relevant signal produced by keratinocytes and fibroblasts, and HGF and bFGF, mainly produced by keratinocytes [36]. Notably, melanogenesis-related signaling pathways may be positively or negatively modulated by several inflammatory factors that finally regulate skin pigmentation. This regulation is very complex as some molecules have a stimulatory effect on melanogenesis, while others show inhibitory effects. In addition, the production of these inflammatory mediators depends on the communication between several epidermal and dermal cells, as well as specific stimuli inducing inflammation [6].

As mentioned above, UV rays represent external stimuli with significant effects on melanogenic signaling pathways, resulting in increased melanin production. Several cellular and molecular processes are involved in the UV–skin reaction. Indeed, UV irradiation induces *MITF* expression and consequently melanogenic gene expression [3,31]. Acquired knowledge shows that UV irradiation influences different skin cell types, including melanocytes, keratinocytes, and dermal fibroblasts. UV-induced melanogenesis has been associated with the release of several molecules, including α-MSH, ACTH, SCF, and ET-1, which regulate the signaling pathways described above [25,36,37,38].

## 4. miRNA Activities and Identification

MicroRNAs (miRNAs) represent a class of non-coding RNAs of approximately 21–23 nucleotides in length. Since their discovery in 1993 [39], miRNAs have been largely studied as essential regulators of gene expression. miRNAs are highly conserved among species and are found in different cell types and organisms, including plants, animals, and viruses [40,41]. It is now estimated that miRNAs target approximately 60% of genes in *humans* and other mammals [42]. Due to their role in mRNA expression regulation, miRNAs are involved in all cellular activities, including proliferation, differentiation, migration, apoptosis, and immune responses, both in normal and pathological conditions [43,44,45,46]. Several mechanisms ensure high stability in miRNAs. Since they can be easily detected in almost all bodily fluids, miRNAs are now considered important biological markers and potential therapeutic molecules [47]. Inside cells, miRNAs derive from long precursor transcripts which give rise to mature miRNAs through a multi-step process and are then incorporated into an RNA–protein complex known as the RNA-Induced Silencing Complex (RISC) [48]. miRNA activity mainly induces gene expression reduction by binding to sequences in the 3′ untranslated region (3′-UTR) of target genes. This can lead to mRNA target degradation, or inhibition of translation and reduction in protein levels [45]. However, miRNA activities show higher complexity as each miRNA can target different genes and several miRNAs can regulate the same gene’s expression. Moreover, the strict interplay between long non-coding RNAs (lncRNAs) and miRNAs contributes to gene expression regulation [40,45,49].

In general, from a methodological point of view, investigations into miRNA functions and roles in a selected process include a preliminary phase of miRNA identification using high-throughput methods such as microarray, followed by target gene identification with a bioinformatics approach. Finally, target genes need to be validated by 3′-UTR reporter assays and analysis conducted on induced cellular effects following transfection experiments with miRNA mimics and antagomiRs [50]. miRNAs are involved in skin development and functions both in normal and pathological conditions [16]. Furthermore, increasing evidence shows that selected miRNAs regulate specific events occurring in the skin, such as melanogenesis, as detailed below [51,52].

### 4.1. miRNA Regulating MITF

Due to the key role of *MITF* as an essential regulator of the melanogenic process, several studies have investigated miRNAs with potential effects on *MITF* expression and consequent regulation of mRNA levels in melanogenic enzymes [3,30,31,53] (Table 1). Some preliminary studies concerning melanogenesis regulation by miRNAs have been performed in fiber-producing animals, such as *alpaca*, due to the contribution of melanin synthesis to coat color and the specific interest of *alpaca* breeders in animal color coat modulation. Differences in miRNA profiles from *alpaca* skins with different colored coats were identified and most differentially expressed miRNAs showed predicted targets involved in pigmentation [54,55]. These results led to further investigation concerning the functional role of selected miRNAs. Data obtained by Zhu Z et al. (2010) demonstrated the functional role of miR-25 in reducing *MITF* mRNA and protein, *TYR*, and *TYRP-1* expression in cultured melanocytes. In addition, an inverse relationship was observed between miR-25 level and coat color. Similarly, in *alpaca* melanocytes, miR508-3p also directly targets *MITF*, binding to the 3′-UTR of the gene. miR508-3p overexpression downregulated *MITF* expression, resulting in a decrease in TYR, TYRP-2, and melanin production [56]. Interestingly, in 2012, Dong C. et al. investigated the role of miR-137, another miR-targeting *MITF*, in a transgenic mice model [57]. Initially investigating melanoma cells lines, where its overexpression downregulated *MITF* [58], it was found that miR-137 also decreased the expression of the MITF protein and its downstream genes in transgenic mice. Notably, miR-137 had an impact on coat color, demonstrating that modulating a specific miR may significantly regulate melanogenesis, at least in animal models [57].

Other studies have identified miRNAs regulating skin pigmentation in *human* melanocytes. Particular interest has been shown in miR-675, another miR which targets *MITF* and was to be found expressed at low levels in the hyperpigmented skin of melasma patients. In melanocytes or keratinocytes derived from melasma patients, miR-675 upregulation decreased TYRP-1 and TYRP-2 expression, whereas its knockdown increased their expression. Interestingly, miR-675 was also identified in exosomes released from keratinocytes into the extracellular environment [5,59,60]. Another miR involved in melanogenesis by binding the 3′-UTR in *MITF* is miR-218, which downregulated TYR, TYRP-1, and DCT mRNA and protein levels, reducing melanin content in immortalized melan-a murine melanocytes. In agreement with these data, miR-218 also suppressed melanogenesis in *human* pigmented skin organotypic culture (OTC) through *MITF* repression. Anti-miR-218 was also found to promote melanogenesis in *human* primary melanocytes [61].

Recently, other miRNAs, including miRNA-183 cluster, miR-340, miR-141-3p, and miR-200a-3p, have been investigated for their role in regulating *MITF* expression and melanogenesis [62]. Results of this research show that the miRNA-183 cluster targets the 3′-UTR of *MITF* in B16 *mouse* melanoma cells. miRNA-183 cluster overexpression decreased *MITF*, *TYR*, *TYRP-1*, and *DCT* expression and melanin production. Conversely, knockdown of the miRNA-183 cluster increased *MITF*, *TYR*, *TYRP-1*, and DCT expression and, consequently, melanin levels. The miR-183 cluster was also involved in regulating the MEK/ERK signaling pathway implicated in cell proliferation and migration, by modulating mitogen-activated protein kinase 1 (MEK1), extracellular regulated protein kinases1/2 (ERK1/2), and CREB expression [62]. miR-340, which was first identified in melanoma cell lines for its ability to bind specifically to the 3′-UTR of *MITF* [64], has been also investigated in immortalized *human* epidermal melanocytes (Pig-1), where it downregulates *MITF* expression and melanin synthesis [63]. Recently, miR-141-3p and miR-200a-3p have been identified as *MITF* regulators [52]. In this study, comparing miRNA expression profiles in B16-4A5 *mouse* melanoma cells which were treated or untreated with α-MSH led to 13 miRNAs being identified as differentially expressed through miRNA array analysis. miR-141-3p, miR-200a-3p, and miR-148a-3p, which target *MITF*, were downregulated in α-MSH-stimulated cells when compared to untreated cells. Furthermore, miR-141-3p and miR-200a-3p overexpression suppressed *MITF* expression and *TYR* activity in B16-4A5 cells. Notably, the inhibitory effect on melanin synthesis was confirmed in a three-dimensional tissue culture model of the *human* epidermis (3D-MHE model) [52].

### 4.2. miRNAs Regulating Other Genes in Melanogenesis

Besides the abovementioned studied group of miRNAs, with significant regulatory roles in *MITF*, other miRNAs are involved in melanogenesis by regulating the expression of other molecular targets, including melanogenic enzymes, transcription factors, or components of the signaling pathways which regulate melanogenesis (Table 2).

Several miRNAs, including miR-450b-5p, miR-1208, miR-326, miR-434-5p, miR330-5p, miR-125, miR-145, and miR-203, have been predicted as targeting *TYR* and most also target other genes, including *MITF* [65,66].

**Table 2:** Other microRNAs involved in melanogenesis.

miRNA	Cell Model	Target Gene	Effect on Melanogenesis	Ref.
miR-434-5p	*Mouse* skin, *human* skin cell cultures	*TYR*	Negative	[65]
miR-330-5p	Melanoma cells, normal *human* melanocytes	*TYR*	Negative	[56,67]
miR-203	Keratinocytes exposed to UV	*Kinesin Superfamily Protein 5b*	Positive	[67]
miR-3196	Keratinocytes exposed to UV	Unknown target gene	Positive	[67]
miR-21a-5p	*Human* melanocytes	*SOX5*	Positive	[68]
miR-145	Murine melan-a melanocytes	*Myo5a*	Negative	[56]
miR-380-3p	*Alpaca* melanocytes	*SOX6*	Negative	[56]
miR-200c	Normal *human* epidermal keratinocytes (NHEK)	*SOX1*	Positive	[69]
miR-27a-3p	*Alpaca* and *Mouse* melanocytes	*Wnt3a*	Negative	[54,70]
miR-379	*Alpaca* melanocytes	*IGF1R*	Negative	[71]
miR-143-5p	*Human* melanocytes	*Myo5a*	Negative	[72]
miR-143-5p	*Alpaca* melanocytes	*TAK1*	Negative	[73]
miR-125b	WM266-4 *human* melanoma cells, MNT1 *human* melanoma cells	*SH3BP4*	Negative	[60]

Wu et al. used a miR-434-5p homologue to target *TYR* in cells cultured in vitro as well as in an animal model and showed efficient melanin synthesis reduction. Similarly, miR-330-5p downregulated *TYR* in melanoma cells and normal melanocytes, inducing depigmentation without affecting cell proliferation [66]. This miR has been also identified in exosomes derived from keratinocytes [56]. In this study, exosomes carrying miR-330-5p caused a decrease in melanin production and *TYR* expression in melanocytes. Similarly, miR-330-5p overexpression in melanocytes confirmed its inhibitory activity on melanogenesis. Several other miRNAs, such as miR-203, which targets *Kinesin Superfamily Protein 5b*, involved in melanosome transfer, and miR-3196, with unknown target genes, have been identified in *human*-derived exosomes from keratinocytes and have shown an ability to increase melanin content in melanocytes [67]. Indeed, exosomes released by Black keratinocytes, as well as Caucasian UV-irradiated keratinocytes, were able to induce increased TYR activity and melanogenic gene expression in melanocytes, at least partly as a result of miR content.

As reported above, several miRNAs which directly target *MITF* are involved in melanogenesis. Conversely, *MITF* expression may be subject to more complex regulation. For example, it has been shown that miR-21a-5p overexpression downregulated the *SOX5* target, as well as β-catenin and CDK2 protein expression, in normal *human* melanocytes. In agreement with *SOX5* involvement in melanogenesis, its downregulation induced an increase in *MITF* and melanogenic enzymes’ expression, with consequent stimulation of melanogenesis [68]. Several other miRNAs have been identified as regulators of SOX transcription factors [56,69,74]. miR-145, which was initially identified as a miR regulated by UV treatment and forskolin in murine melan-a melanocytes, significantly modulates several genes involved in pigmentation. miR-145 overexpression or downregulation reduced or increased the expression of several genes involved in melanin biosynthesis, such as *SOX9*, *MITF*, *TYR*, and *TYRP-1* [74,75], and in melanosome transfer, such as *Myosin Va* (*Myo5a*), *Rab27a*, and *fascin1* (*Fscn1*), respectively [76]. Notably, miR-145 targeted the 3′-UTR binding site of *Myo5a*, a molecular motor involved in the intracellular trafficking of vesicles and organelles. A further miR that binds an SOX family component is miR-380-3p, which targets *SOX6* by binding to the 3′-UTR region. In *alpaca* melanocytes, miR-380-3p overexpression downregulated *SOX6* and increased β-catenin. Differently from studies indicating that β-catenin induces *MITF* transcription, in this study, miR-380-3p overexpression caused decreased mRNA levels of melanin-related genes including *MITF*, *TYR*, *TYRP-1*, and *DCT*, suggesting other *SOX6* activities in *MITF* regulation [56].

Recently, miR-200c has been identified as a *SOX1* regulator. Specifically, miR-200c induced *SOX1* downregulation by direct binding to the *SOX1* 3′-UTR, leading to intranuclear β-catenin upregulation and increased expression of *MITF*-dependent gene expression as well as melanogenesis. These effects were induced in normal *human* epidermal melanocytes (NHEM) treated with exosomes containing miR-200c, produced by normal *human* epidermal keratinocytes (NHEK). Notably, miR-200c was found at low levels in exosomes derived from keratinocytes in vitiligo lesions compared to exosomes from NHEK, with a consequent inhibition in melanin production [69]. Some miRNAs have also been involved in modulating the extracellular ligands which regulate melanogenic signaling pathways. An example is miR-27a-3p, which directly targets *Wnt3a*, a component of the Wnt signaling pathway. miR-27a-3p was found to be expressed at higher levels in *alpaca* white skin compared to brown skin [54] and was similarly related to skin color in mice [70]. Furthermore, its functional role in downregulating *Wnt3a*, and thus inhibiting β-catenin and melanogenesis, was confirmed in *mouse* melanocytes transfected with miR-27a-3p [70]. In addition, miR-379 was shown to modulate *insulin-like growth factor receptor I* (*IGFR1*) in *alpaca* melanocytes. Insulin-like growth factor 1 (IGF1), mainly produced in dermal cells, has been shown to improve melanogenesis through the cAMP pathway. Likewise, miR-379 overexpression reduced melanogenesis by inhibiting the cAMP response element (CRE)-binding protein (CREB)/(*MITF*) pathway in *alpaca* melanocytes [71].

Finally, miRNAs involved in the regulation of melanosome processing and transfer have been also identified. Among them, miR-143-5p targets *Myo5a*, which belongs to the complex *Rab27a/MLPH/Myo5a* that connects melanosomes to the actin cytoskeleton in *human* melanocytes [72]. In a more recent study, Qi S. et al. inhibited miR-143-5p expression using Short Tandem Target Mimics (STTMs) in order to evaluate the functional role of miR-143-5p. As a consequence, increased melanogenic gene expression, including *MITF*, TYR, and TYRP-1, MLPH, and Rab27, and melanin production were observed [77]. This result indicates the effective possibility of modulating melanin biosynthesis by a miRNA-regulating approach. In addition, miR-143-5p overexpression induces a decrease in *TGF-β-activated kinase 1* (*TAK1*) expression with consequent effects on melanocyte migration and proliferation and *MITF* downregulation in *alpaca* melanocytes. Therefore, miR-143-5p may regulate melanogenesis by modulating different gene targets [73]. By means of a bioinformatic approach, followed by experimental validation, miR-125b has also been shown to directly target *SRC homology 3 domain-binding protein 4* (*SH3BP4*), a gene regulated by *MITF*, which may act by controlling melanogenic enzymes’ distribution to melanosomes or the mTOR signaling pathway. Indeed, when miR-125b was overexpressed in WM266-4 and MNT1 *human* melanoma cells, decreased levels of the melanogenic enzymes TYR and DCT were observed, although these genes are not direct miR-125b targets [78].

### 4.3. miRNA Regulated by UV Rays

UV rays can modify intracellular functions in different ways: by directly or indirectly damaging DNA through reactive oxygen species (ROS), inducing apoptosis, cell cycle arrest, and carcinogenesis. The expression profiles of the miRNAs involved in these events are modified by UV irradiation [79,80,81]. Among the UV-induced effects, it is well known that there is a correlation between UVB irradiation and melanogenesis stimulation [1]. miRNA profiling in irradiated *mouse* melanocytes has been helpful in identifying miRNAs involved in melanogenesis, such as miR-145 [74]. Furthermore, several miRNAs targeting different pigmentation genes have been involved in UV ray effects, including miR-145, miR-137, miR-148, and miR-25 [57,82,83].

miR-340 upregulation was identified in pigmented cells treated with UVB irradiation among miRNAs related to UV irradiation. Studies have shown that miR-340 significantly represses RhoA protein expression and stimulates melanosome transport [81]. Recently, the functional role of miR-340 has been further investigated in immortalized *human* epidermal melanocytes (Pig-1), confirming that its expression is modulated by UV and that it downregulates *MITF* expression and melanin synthesis. Notably, miR-340 mimics decreased melanin content in irradiated cells [63]. A further example of a miR potentially being involved in UV-induced pigmentation modulation is miR-21. The latter has been largely investigated in melanoma and shown to be increased by UV radiation in melanocytes, keratinocytes, and fibroblasts [84]. In *mouse* skin melanocytes, miR-21 has been shown to enhance *MITF* expression by targeting *SOX5* [85]. Inversely, miR-21a-5p has been observed with different levels of activity in UV-irradiated A375.S2 *human* melanoma and B16F10 melanoma *mouse* cells. Indeed, in these UV-treated cells, increased melanin content was associated with an increase in α-MSH expression and reduced EGFR and Akt phosphorylation. miR-21 overexpression negatively modulated these UV-induced effects [86]. Interestingly, this miR has been also involved in communication between melanocytes and keratinocytes. Extracellular vesicles derived from UVA-exposed melanocytes modified keratinocyte behavior by inducing miR-21 upregulation and TGF-β and IL-6/STAT3 signaling pathway activation [87]. UVA and UVB rays also induce changes in miRNA produced by keratinocytes [88,89]. Accumulating evidence indicates that exosomes and exosomal miRNAs represent an effective means of communication for melanocytes and keratinocytes [56]. Notably, in Caucasian *human* keratinocytes, UV rays induce the release of miR, such as hsa-miR-3196, which may stimulate melanin synthesis in melanocytes [67]. Recently, miR-675 production by *human* keratinocytes has been shown and its role as a paracrine regulator of melanogenesis has been confirmed in vitro, indicating increased exosomal miR-675 in cells irradiated with a 585 nm light emitting diode (LED) and its ability to attenuate melanogenesis in melanocytes [90].

### 4.4. Common miRNAs in Melanogenesis and Melanomagenesis

Several miRNAs regulating melanogenesis play an important role in melanoma [91]. For instance, miR-340, miR-218, and miR-137, which inhibit melanogenesis, have a tumor suppressor role during melanomagenesis by negatively regulating *MITF* expression [58,61,64,92]. Other miRNAs which regulate melanogenesis, including miR-379, -200c, -203, -200a-3p, -125b, -183, -508-3p, -675, -143-5p, -141-3p, -145, and -330-5p, can act as tumor suppressors in melanomagenesis, although the target genes may differ from those identified in melanogenesis. On the contrary, miR-21a-5p, -25, and -27a-3p have shown an oncogenic role, being upregulated in metastatic melanoma and during tumor progression [93,94].

Interestingly, some miRNAs involved in both melanogenesis and melanomagenesis, such as miR-21a-3p, -25, -3196, -145, -137, -148, and -675 are induced by UV rays, the main environmental risk factor for melanoma [79,80,81,95].

## 5. Conclusions

Melanogenesis is the main event involved in regulating skin color and plays a pivotal role in protection from UV rays, as it limits UV damage and genotoxic effects [1]. Melanogenesis dysregulation is observed in several skin pigmentation disorders and has been related to skin cancerogenesis [5]. Therefore, melanogenesis control and regulation play a significant role in skin pathophysiology. Melanogenesis involves different cell types and is regulated by several external and internal factors, including UV rays, hormones, growth factors, and cytokines, which act autocrinally and paracrinally by modulating several cellular signaling pathways [26]. Here, studies showing that several miRNAs participate in the complex regulation of melanogenesis have been reviewed. These data show the significant impact and functional role of miRNAs in the melanogenic process. miRNAs have been identified using different approaches, including miRNA profile evaluation, bioinformatic analysis, and experimental investigation of cellular effects induced by miRNA regulation [48]. Above all, melanocytes and melanoma cell lines were used as they represent the key cell players in melanogenesis. Conversely, since the complex regulation of melanogenesis is based on the strict interplay between melanocytes and keratinocytes, as well other skin cells [6,10,36,96], some studies have investigated miRNAs expressed in keratinocytes, providing relevant information concerning the fine cellular and molecular regulation involved in this process.

Overall, most identified miRNAs, including miR-25, miR508-3p, miR-137, miR-218, miR-183, miR-340, miR-141-3p, and miR-200a-3p, target *MITF*, leading to a decrease in its gene expression and consequent melanogenesis inhibition [52,58,61,62,63,73]. These miRNAs include miR-137, which was able to modulate coat color in transgenic mice [57]. This result appears to be of particular interest as it demonstrates that modulating miRNA expression may be a way to significantly regulate melanogenesis in vivo. In *humans*, some miRNAs have also been associated with changes in skin color and pigmentation dysfunction, including miR-675 [60], which was expressed at a low level in the hyperpigmented skin of melasma patients, and miR-200c, which was found at low levels in exosomes obtained from vitiligo patients [69]. In addition, miRNAs can regulate melanogenesis by modulating the expression of genes coding for extracellular mediators such as *wnt3a* [54,70]; membrane receptors such as IGF-IR [71]; intracellular components of the melanogenic signaling pathways, such as *SOX* transcription factors; or molecules involved in melanosome transfer, such as *Myo5a*, *Rab27a*, and *Fscn1* [7,56,69,74,76]. Notably, several miRNAs, including miR-675, miR-330-5p, miR-203, miR-3196, and mir-200c, have been identified in exosomes derived from keratinocytes and their functional role in the regulation of melanocyte activities and melanogenesis has been proven [5,59,60,67,69,97]. This identifies miRNAs as important extracellular molecular mediators, thus increasing our understanding of the close correlation between skin cell types which regulate melanogenesis. In this context, further knowledge on the potential contribution of dermal fibroblasts, as well as inflammatory skin cells, is needed [6,69].

Due to the essential role of UV rays in skin pigmentation, several studies have also investigated miRNAs modulated by UV irradiation, showing that miRNA levels can be changed by UV radiation [79,80,81]. These miRNAs deserve particular attention due to their involvement in skin hyperpigmentation induced by UV rays, and potentially in skin carcinogenesis [84]. Indeed, here, we have reported that several miRNAs can be involved in both melanogenesis and melanomagenesis. Knowledge on miRNAs physiologically involved in melanogenesis is extremely important; alterations in melanocyte miRNA signatures might serve as diagnostic markers for melanoma progression and may help to plan targeted therapies in the future [92].

Several causes and events can lead to altered pigmentation by regulating inflammatory reactions. Therefore, while the role of inflammation in modulating cell behavior and the signaling pathways involved in skin pigmentation is beginning to be elucidated [6], further investigation concerning miRNAs involved in skin inflammation might be helpful. In addition, based on increasing knowledge on the relationship between different classes of non-coding RNAs, new relevant information is expected from studies concerning the interaction and reciprocal regulation of miRNAs and other non-coding RNA classes [14,98]. In conclusion, increasing evidence shows the involvement of miRNAs in melanogenesis and skin pigmentation disorders. Elucidation of the role of melanogenic signaling pathways, as well as intercellular communication, might be improved by a deeper understanding of miRNAs’ activities in controlling and regulating gene targets and melanogenesis and by identifying novel miRNAs. Encouraging data are beginning to show the effective role of miRNAs in the regulation of skin pigmentation, confirming their role in more complex in vitro models such as three-dimensional skin organotypic culture models [61]. Notably, recent topical transfection of miR-141-3p and miR-200a-3p to three-dimensional reconstructed *human* skin tissue inhibited α-MSH-induced melanin biosynthesis with similar efficiency as obtained by arbutin, a well-known depigmentating molecule [52].

Further investigations using advanced in vitro models, such as co-culture of skin cells and organotypic skin culture, are to be encouraged in order to better clarify the essential role of miRNAs in melanogenesis, taking into account the strict correlation among cells that participate in the process [99]. Epigenetic regulation of melanogenesis provides a novel insight into the melanogenesis mechanism and suggests potential therapeutic approaches for the unsolved problem of skin pigmentation disorders [7], based on the use of miRNA mimics and antagomiRs [52,100].

## Figures and Tables

**Table 1:** microRNAs regulating MITF in melanogenesis.

miRNA	Cell Model	Target Gene	Effect on Melanogenesis	Ref.
miR-25	*Alpaca* melanocytes	*MITF*	Negative	[56]
miR508-3p	*Alpaca* melanocytes	*MITF*	Negative	[56]
miR-137	*Alpaca* melanocytes	*MITF*	Negative	[58]
miR-675	Melanocytes of melasma patients, keratinocytes of melasma	*MITF*	Negative	[5,59,60]
miR-218	Melan-a murine melanocytes, *human* skin OTC	*MITF*	Negative	[61]
miR-183	B16 melanoma cells	*MITF*	Negative	[62]
miR-340	*Human* epidermal melanocytes (Pig-I)	*MITF*	Negative	[63,64]
miR-200a-3p	B16-4A5 melanoma cells	*MITF*	Negative	[52]
miR-148a-3p	B16-4A5 melanoma cells	*MITF*	Negative	[52]
miR-141-3p	B16-4A5 melanoma cells	*MITF*	Negative	[52]

## Abbreviations

3′-UTR	three prime untranslated region
3D-MHE model	three-dimensional tissue culture model of *human* epidermis
ACTH	adrenocorticotrophic hormone
AKT	protein kinase B
bFGF	basic fibroblast growth factor
bHLH-ZIP	basic helix-loop-helix leucine zipper
cAMP	cyclic adenosine monophosphate
CDK2	cyclin-dependent kinase 2
CREB	cAMP-responsive element binding protein
DAG	diacyglycerol
DCT	dopachrome tautomerase
ERK1/2	extracellular regulated protein kinases1/2
ETRB	endothelin type B receptor
Fscn1	fascin1
FZD	frizzled receptor
GPCRs	G protein-coupled receptors
GSK3β	glycogen synthase kinase 3β
HGF	hepatocyte growth factor
IGF-1R	insulin-like growth factor receptor I
IL-6	interleukin-6
IP3	inositol triphosphate
KGF	keratinocyte growth factor
L-DOPA	dihydroxyphenylalanine
LED	light emitting diode
LEF-1	lymphoid enhancer-binding factor 1
LEF-TCF	lymphoid enhancing factor-1/T-cell factor
lncRNA	long non-coding RNAs
MAPK	mitogen-activated protein kinase
MC1R	melanocortin 1 receptor
MEK1	mitogen-activated protein kinase 1
miRNAs	microRNAs
MITF	microphthalmia-associated transcription factor
MLPH	melanophilin
MYO5A	myosin VA
NHEK	normal *human* epidermal keratinocytes
NHEM	normal *human* epidermal melanocytes
NO	nitric oxide
OTC	skin organotypic culture
PAX3	paired box family of transcription box 3
PGE2	prostaglandin E2
PI3K	phosphoinositide-3 kinase
Pig-1	immortalized *human* epidermal melanocytes
PIP2	phosphatidylinositol 4,5-bisphosphate
PKA	protein kinase A
PLCγ	activated phospholipase Cγ
POMC	proopiomelanocortin
RISC	RNA-Induced Silencing Complex
RNA	ribonucleic acid
ROS	reactive oxygen species
RSK	ribosomal S6 kinase
SCF	stem cell factor
SH3BP4	SRC homology 3 domain-binding protein 4
SOX	sex-determining region Y-box
SOX1	sex determining region Y-box 1
SOX10	sex determining region Y-box 10
SOX5	sex determining region Y-box 5
SOX6	sex determining region Y-box 6
SOX9	sex determining region Y-box
STAT3	signal transducer and activator of transcription 3
STTMs	short tandem target mimic
TAK1	TGF-β-activated kinase 1
TGF-β	transforming growth factor-β
TYR	tyrosinase
TYRP-1	tyrosinase-related protein 1
TYRP-2	tyrosinase-related protein 2
UV	ultraviolet
UVA	ultraviolet A
UVB	ultraviolet B
WNT3A	Wnt family member 3A
α-MSH	α-melanocyte stimulating hormone
